# Multi-Scale Convolutional Attention and Structural Re-Parameterized Residual-Based 3D U-Net for Liver and Liver Tumor Segmentation from CT

**DOI:** 10.3390/s25061814

**Published:** 2025-03-14

**Authors:** Ziwei Song, Weiwei Wu, Shuicai Wu

**Affiliations:** 1 Department of Biomedical Engineering, College of Chemistry and Life Sciences, Beijing University of Technology, Beijing 100124, China; songziwei1121@163.com; 2 College of Biomedical Engineering, Capital Medical University, Beijing 100069, China

**Keywords:** liver and tumor segmentation, 3D UNet, multi-scale convolutional attention, structural re-parameterization, multi-feature extraction

## Abstract

Accurate segmentation of the liver and liver tumors is crucial for clinical diagnosis and treatment. However, the task poses significant challenges due to the complex morphology of tumors, indistinct features of small targets, and the similarity in grayscale values between the liver and surrounding organs. To address these issues, this paper proposes an enhanced 3D UNet architecture, named ELANRes-MSCA-UNet. By incorporating a structural re-parameterized residual module (ELANRes) and a multi-scale convolutional attention module (MSCA), the network significantly improves feature extraction and boundary optimization, particularly excelling in segmenting small targets. Additionally, a two-stage strategy is employed, where the liver region is segmented first, followed by the fine-grained segmentation of tumors, effectively reducing false positive rates. Experiments conducted on the LiTS2017 dataset demonstrate that the ELANRes-MSCA-UNet achieved Dice scores of 97.2% and 72.9% for liver and tumor segmentation tasks, respectively, significantly outperforming other state-of-the-art methods. These results validate the accuracy and robustness of the proposed method in medical image segmentation and highlight its potential for clinical applications.

## 1. Introduction

Primary liver cancer is the seventh most common cancer worldwide, posing a significant threat to human life and health. According to the latest statistics, liver cancer ranks as the second leading cause of cancer-related deaths and the fourth most prevalent cancer in China [1]. Computed tomography (CT) is currently one of the most widely used diagnostic methods for liver lesions. CT imaging provides essential information regarding the morphology, quantity, location, and boundaries of liver tumors, making the effective segmentation of liver tumor regions based on CT images highly valuable for clinical applications. However, challenges remain because of the similarity in grayscale values between the liver and surrounding organs in CT images, significant inter-patient variability, and the fact that liver tumors can appear at any location with indistinct boundaries. Consequently, three-dimensional (3D) segmentation of the liver and liver tumors remains a highly complex task [2].

Before 2016, most automatic liver and tumor segmentation methods relied on traditional machine learning techniques. These approaches included methods based on the shape and geometric priors, intensity distribution, and spatial context for liver segmentation [3,4,5,6,7], as well as thresholding, spatial regularization, or local features combined with learning algorithms for tumor segmentation [8,9,10,11,12]. However, these methods were characterized by low efficiency, limited robustness, and a reliance on the manual adjustment of numerous parameters, often requiring expert experience.

Since the first related paper was published at MICCAI in 2016, deep learning methods have gradually become the preferred approach for liver and tumor segmentation tasks. The application of deep learning methods in medical image segmentation has become increasingly widespread. Convolutional neural networks (CNNs), fully convolutional networks (FCNs), U-Net, and their derivative architectures have demonstrated exceptional performance in a variety of segmentation tasks [13,14]. Christ et al. [15] were the first to apply the 3D UNet architecture for this purpose, introducing a cascaded segmentation strategy combined with 3D conditional random fields for refinement. Subsequently, numerous deep learning approaches have been developed and evaluated using the publicly available LiTS dataset [16]. The accessibility of this dataset has facilitated the proposal of various novel deep learning solutions for liver and tumor segmentation. Among these, U-Net-based architectures have been extensively adopted and modified to enhance segmentation performance.

For example, Li et al. [17] proposed the H-Dense UNet with hybrid dense connections, which effectively optimized in-slice features and 3D contextual information, resulting in improved segmentation accuracy. Dey et al. [18] designed a cascaded model that utilized a 2D deep convolutional network to segment the liver and larger tumor regions, followed by a 3D network to handle smaller tumors, thereby enhancing the segmentation accuracy in complex scenarios. Han et al. [19] introduced a 2.5D deep CNN that integrates long-range connections from U-Net with short-range residual connections from ResNet, creating a deeper and wider architecture while retaining compatibility with 3D information. Gruber et al. [20] compared the following two segmentation schemes: direct end-to-end segmentation using a 2D U-Net and a cascaded approach employing two 2D U-Nets. In the latter approach, the liver region was segmented first, and the region of interest (ROI) was extracted for subsequent tumor segmentation, demonstrating a superior segmentation performance. Bi et al. [21] developed a novel cascaded ResNet architecture with multi-scale fusion, achieving excellent boundary segmentation between the liver and lesions. Kaluva et al. [22] utilized a densely connected, full CNN (DenseNet) for fully automated, two-stage cascaded segmentation of the liver and tumors. Deng et al. [23] combined a 3D densely connected network with a level set method to dynamically adjust segmentation parameters and initialize local segmentation windows, enabling precise tumor boundary detection and segmentation. Additionally, Liu et al. [24] proposed a spatial feature fusion convolutional network that efficiently segmented both the liver and tumors from CT images. Zhu et al. [25] introduced a novel neural network architecture, an FM-FCN, which excels in feature extraction. Sun et al. [26] proposed RHEU-Net, a multi-scale liver tumor segmentation method based on residual modules and hybrid attention mechanisms, significantly improving segmentation accuracy. Ma et al. [27] presented the “Segment Anything” framework, a deep learning model for medical images that boasts zero-shot segmentation capabilities, particularly excelling in the segmentation of small tumors or low-contrast regions.

These studies introduced techniques, such as multi-scale modeling, cascaded segmentation, and dense connections, offering efficient solutions for liver and tumor segmentation while significantly improving the segmentation performance. Notably, the nnU-Net, proposed during the MICCAI 2018 LiTS challenge [28], has been recognized as one of the top-performing methods for 3D image segmentation tasks. Since then, 3D deep learning models have gained widespread popularity, frequently outperforming 2.5D and 2D models without the need for more complex preprocessing steps.

This paper introduces the ELANRes-MSCA-UNet model, an enhancement of the 3D U-Net architecture, which integrates the ELANRes residual module and the MSCA multi-scale convolutional attention module. A two-stage learning strategy is employed, wherein the liver region is segmented first, followed by tumor segmentation within the liver. This approach effectively enhances the accuracy of small-target segmentation and reduces false positives. By combining multi-scale modeling and boundary optimization, the model strikes a balance between global feature extraction and local detail refinement, leading to significant improvements in the segmentation accuracy and robustness. The remainder of this paper is structured as follows: Section 2 provides a detailed description of the proposed method; Section 3 presents the experimental design and analysis of the results; and Section 4 concludes the paper.

## 2. Methods

### 2.1. Methodology Workflow

**Figure 1:**
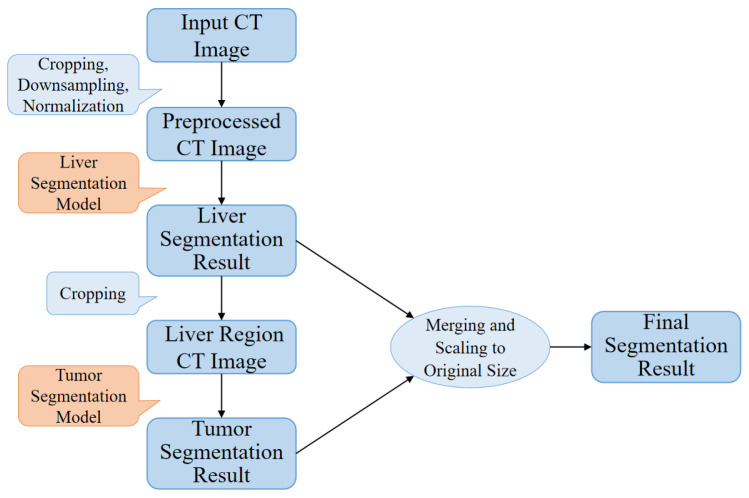
Liver and liver tumor segmentation flow chart.

This paper proposes a high-precision liver and liver tumor segmentation method based on a two-stage strategy. The workflow, as illustrated in Figure 1, consists of the following four main components: data preprocessing, liver segmentation (first stage), tumor segmentation (second stage), and post-processing. The details are as follows: (1) Data Preprocessing: Threshold segmentation and morphological operations are initially applied to generate a body mask from the original CT images, which is used to crop the effective abdominal region. The cropped images are then downsampled and normalized, with the spatial resolution uniformly adjusted to 0.5 mm × 0.5 mm × 1.0 mm (where the x- and y-axes are halved from the original resolution, while the *z*-axis remains unchanged). The CT intensity values are truncated to the range of −200 to 200 HU and normalized to a 0–1 range using a normalization factor of 200.0, providing standardized inputs for training the deep neural networks. (2) Liver Segmentation (first stage): The proposed ELANRes-MSCA-UNet network is employed to accurately locate and segment the liver region from abdominal CT images, laying the foundation for subsequent tumor segmentation. This stage aims to minimize interference from non-liver tissues, ensuring a reliable liver region mask. (3) Tumor Segmentation (second stage): Using the liver mask generated in the first stage, tumor segmentation is confined to the liver region. By extracting tumor features within this specific area, interference from other tissues is eliminated, reducing tumor localization complexity and improving segmentation accuracy. Tumor segmentation also utilizes the ELANRes-MSCA-UNet network, which is independently trained to optimize tumor segmentation performance. (4) Post-Processing: The largest connected component method is applied to the liver segmentation results to remove false-positive regions, ensuring the reliability of the segmentation output. The liver and tumor segmentation results are then combined, restored to the spatial position of the original CT image, and interpolated to match the original image size, producing the final prediction output.

This two-stage segmentation strategy optimizes liver and tumor segmentation tasks step by step, significantly improving the segmentation accuracy and robustness. The decision to use a two-stage approach stems from the fact that liver segmentation in the first stage acts as a preprocessing step, simplifying the subsequent tumor segmentation. By isolating the liver first, the model can focus solely on the tumor in the second stage, reducing interference from surrounding non-liver tissues. This structured method creates a more controlled environment for tumor segmentation, allowing the model to more accurately detect and delineate tumor boundaries, especially in challenging cases involving small or low-contrast tumors.

Furthermore, the two-stage approach helps reduce false positives, which are common in single-stage methods. In a single-stage process, the model must segment both the liver and tumor simultaneously, increasing the risk of errors in tumor localization due to the surrounding tissue. By segmenting the liver first, the two-stage method simplifies the task, ensuring more accurate tumor localization and an overall improved segmentation performance.

### 2.2. Proposed ELANRes-MSCA-UNet Network

To address the challenges of medical image segmentation, including complex target morphology, multi-scale features, and blurred boundaries, this paper proposes a novel network architecture called ELANRes-MSCA-UNet. Building upon the classical 3D U-Net and inspired by the design principles introduced by Ding et al. [29,30,31], the network incorporates a structurally re-parameterized residual module (ELANResBlock) and a multi-scale convolutional attention module (MSCABlock) into the 3D U-Net framework. The decision to adopt the ELANRes and MSCA modules, instead of other commonly used methods like SE blocks or standard residual connections, stems from their ability to specifically enhance segmentation performance. The ELANResBlock improves feature extraction by employing structural re-parameterization, thus optimizing resource utilization compared to standard residual connections. In addition, the MSCA module integrates multi-scale convolutions and attention mechanisms, enabling the network to capture global contextual information more effectively. This is particularly advantageous for accurately segmenting small or low-contrast tumors. In contrast, SE blocks, while useful for recalibrating feature channels, do not capture the same level of global spatial information, making them less suitable for complex segmentation tasks. These enhancements significantly improve the network’s feature extraction capabilities and segmentation accuracy, while maintaining high computational efficiency in tackling complex medical image segmentation tasks.

**Figure 2:**
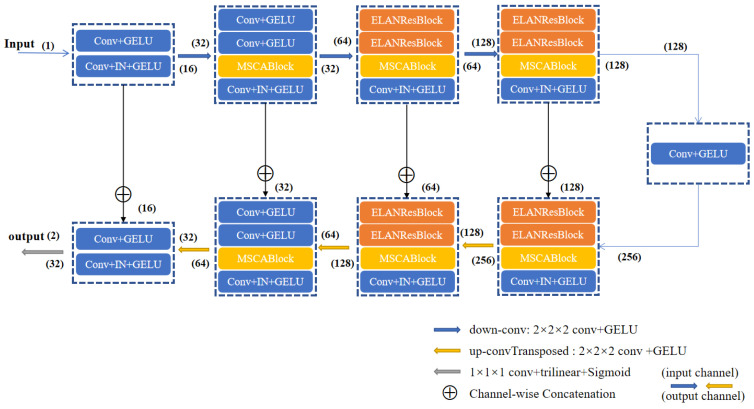
The ELANRes-MSCA-UNet’s network architecture. The number above the arrow represents the input channels, and the number below the arrow represents the output channels.

As shown in Figure 2, the ELANRes-MSCA-UNet employs a 3D U-Net architecture, consisting of the following four primary components: an encoder, a decoder, a bottleneck layer, and skip connections. The encoder progressively extracts multi-scale 3D features using 3D convolutions and downsampling, effectively capturing spatial relationships across all three dimensions. The decoder restores spatial resolution through upsampling and further refines segmentation results by integrating multi-scale 3D features from the encoder. The bottleneck layer, serving as a critical bridge between the encoder and decoder, fuses multi-scale 3D contextual features and utilizes 3D convolution operations to provide high-level semantic information, enhancing the model’s understanding of spatial relationships within medical images. Skip connections facilitate the transfer of high-resolution 3D features from the encoder to the decoder through channel-wise concatenation, mitigating information loss caused by downsampling and maintaining spatial accuracy across the three dimensions. Finally, the network generates high-resolution segmentation maps via a 1 × 1 convolution followed by trilinear upsampling, preserving fine 3D spatial details and integrating semantic information across multiple scales, thereby significantly improving segmentation accuracy.

**Figure 3:**
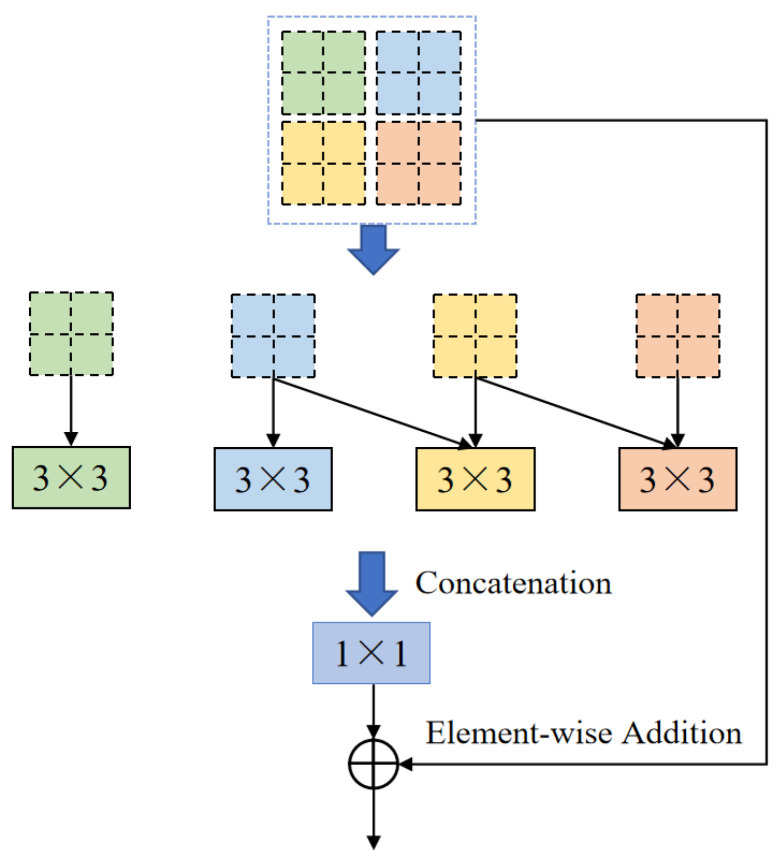
Core structure of the ELANRes module.

One of the core modules of ELANRes-MSCA-UNet, the ELANResBlock (shown in Figure 3), extracts diverse feature representations using parallel 3 × 3 convolutions and employs 1 × 1 convolutions for channel fusion. This module incorporates structural re-parameterization technology, which enhances feature diversity during the training phase while collapsing the parallel convolutions into an equivalent single convolution during the inference phase. By increasing the feature representation capacity and minimizing network parameters, the ELANResBlock effectively optimizes the overall performance of the network. Additionally, the module excels at boundary recognition, especially in handling blurred boundaries between the liver and tumor. The structural re-parameterization allows the network to more accurately delineate complex boundaries, improving the segmentation accuracy, particularly when distinguishing among regions with similar intensities, such as the liver and tumors.

**Figure 4:**
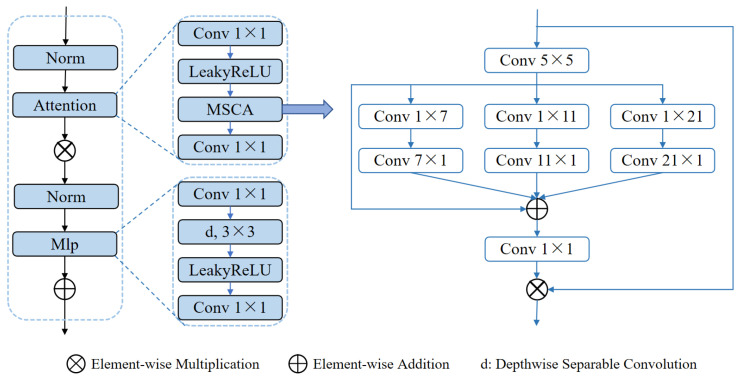
Core structure of the MSCA module.

Another core module, the MSCABlock (shown in Figure 4), captures rich contextual information through multi-scale convolutions and attention mechanisms. The module begins by normalizing the input features and extracting multi-level features using convolutional kernels of varying scales (5× 5, 1 × 7 + 7 × 1, 1 × 11 + 11 × 1, 1 × 21 + 21 × 1). These features are subsequently fused via element-wise addition and refined through 1 × 1 convolutions for channel optimization. An embedded residual connection dynamically focuses on key feature regions, while a multi-layer perceptron (MLP) further enhances feature representation. Additionally, the module applies a 3D-to-2D transformation to exploit the efficiency of 2D convolutions, preserving both global and local multi-scale information. This design significantly enhances the segmentation performance in complex scenarios. In particular, through multi-scale convolutions, the module improves the network’s ability to capture small targets, such as small tumors, which might otherwise be overlooked in a single-scale approach. The attention mechanism further improves the network’s focus on critical regions, reducing background noise interference and improving segmentation precision for small targets. By enhancing both global context and local detail, the MSCABlock effectively handles the segmentation of small and subtle targets, demonstrating notable advantages in scenarios where target regions are small, complex, or have indistinct boundaries.

By integrating the ELANResBlock and MSCABlock, the ELANRes-MSCA-UNet strikes an effective balance between feature extraction, multi-scale contextual modeling, and computational efficiency. This architecture exhibits an exceptional segmentation performance, offering a novel and robust solution for medical image segmentation tasks.

### 2.3. Loss Function Combination and Deep Supervision Mechanism

To improve the model’s performance in segmentation tasks, particularly in addressing class imbalance and small-target segmentation challenges, multiple loss functions were designed and combined. By employing a weighted combination of these loss functions, complementary advantages across different tasks are achieved, effectively optimizing the overall segmentation performance. These functions are specifically tailored to enhance segmentation accuracy, improve boundary recovery, and increase the model’s sensitivity to fine-grained targets.

Firstly, the Dice loss function was introduced to evaluate the overlap between the predicted results and the ground truth, effectively enhancing the overall segmentation accuracy. To address class imbalances, the Tversky loss function was designed, assigning different weights to false positives and false negatives, thereby demonstrating strong adaptability to small-target segmentation scenarios. The binary cross-entropy (BCE) loss function significantly enhances the model’s accuracy in binary classification tasks by calculating pixel-level cross-entropy, particularly in scenarios involving blurred boundaries or low contrast. It contributes to enhancing the stability and robustness of the segmentation results. To enhance the model’s performance in complex segmentation tasks, two composite loss functions (hybrid loss) were designed. HybridLoss1 integrates Dice loss with BCE loss to balance the pixel-level classification accuracy and region segmentation performance. HybridLoss2 integrates Tversky loss with BCE loss to optimize the model’s performance in scenarios involving class imbalance and complex segmentation, especially when handling small targets and complex boundaries. The formulas of different loss functions are shown in Table 1.

Here, Intersection refers to the intersection of the predicted results and the ground truth, while Union represents their union. $\epsilon =1\times 10^{-5}$ is a smoothing term; α=0.3 and β=0.7 are the weights used to control the penalties for false positives and false negatives, respectively; y denotes the ground truth label, where $y_{i}\in \{0,1\}$, and ${\hat{y}}_{i}$ represents the model’s predicted probability, with ${\hat{y}}_{i}\in [0,1]$; *N* represents the total number of pixels in the sample, and $y_{i}$ indicates the true label of the *i*-th pixel; and λ=0.5 is a weight parameter that balances the influence of the two losses.

During training, the network’s multiple outputs correspond to feature maps at different scales, with each output optimized using its respective loss function. The specific loss calculation is as follows:

(1) TotalLoss=loss3+α⋅(loss0+loss1+loss2)

Here, *loss*3 represents the loss for the final output, which typically has the highest resolution, while *loss*0, *loss*1, and *loss*2 correspond to the losses of low-resolution outputs. Using a deep supervision mechanism, higher weights (*α* = 0.4) are assigned to the losses of low-resolution feature maps during the early training stage. These weights are gradually reduced in later stages, allowing the model to rely primarily on high-resolution outputs during the final stage. The weight, *α*, decays periodically during the training process as follows:

(2) α=α×0.8

This design accelerates model convergence and improves its performance in multi-scale segmentation tasks.

### 2.4. Evaluation Metrics

To comprehensively evaluate the model’s performance in liver and liver tumor segmentation tasks, multiple metrics were selected from various perspectives. These include the Dice score (*Dice*), average symmetric surface distance (*ASD*), relative volume difference (*RVD*), precision, and recall. Together, these metrics offer a holistic assessment of the segmentation performance, addressing accuracy, geometric shape, volume discrepancy, and classification capability. For liver segmentation tasks, *Dice* and ASD are used as evaluation metrics, whereas all five metrics are applied to provide a comprehensive evaluation of liver tumor segmentation performance.

The *Dice* score measures the overlap between the predicted results and ground truth, with higher values indicating more accurate segmentation. The *ASD* evaluates the geometric deviation between the predicted and ground truth contours, where smaller values reflect segmentation boundaries that are closer to the ground truth. The *RVD* quantifies the relative difference between the predicted and actual volumes, with values closer to 0 indicating better consistency between the predicted and true volumes. Precision represents the proportion of correctly identified positive samples within the predictions, while recall measures the proportion of true positive samples that are correctly identified.

The formulas for calculating these metrics are presented in Equations (3)–(8), comprehensively addressing all critical aspects of segmentation performance and providing a reliable foundation for model evaluations.

(3) $$Dice(A,B)=\frac{2|A\cap B|}{\left|A|+|B\right|}$$

(4) $$ASD(A,B)=\frac{1}{\left|S(A)|+|S(B)\right|}\left(\sum_{s_{A}\in S(A)}d(s_{A},S(B))+\sum_{s_{B}\in S(B)}d(s_{B},S(A))\right)$$

(5) $$RVD(A,B)=\frac{\left|B|-|A\right|}{\left|A\right|}$$

(6) $$precision=\frac{TP}{TP+FP}$$

(7) $$recall=\frac{TP}{TP+FN}$$

(8) $$d(v,S(A))=\min_{s_{A}\in S(A)}||v-s_{A}||$$

Here, *A* and *B* represent the predicted result and ground truth regions, respectively; *TP*, *TN*, *FP*, and *FN* denote true positives, true negatives, false positives, and false negatives, respectively; *S*(*A*) and *S*(*B*) represent the boundary sets of the predicted region *A* and the ground truth region *B*, respectively; and *d*(*v,S*(*A*)) represents the shortest distance from a point *v* to the boundary *S*(*A*) of region *A*.

## 3. Experiments and Results

### 3.1. Data and Implementation

We utilized the MICCAI 2017 LiTS Challenge dataset to train the automatic segmentation model [32], and the trained model was tested on both the LiTS dataset and the 3Dircadb (https://www.ircad.fr/research/data-sets/liver-segmentation-3d-ircadb-01/ (accessed on 1 March 2025)) dataset. The LiTS dataset comprises abdominal CT images collected from seven different medical centers, including a total of 131 cases. Each case was manually annotated by radiation oncologists with liver and liver tumor contours serving as the ground truth. The pixel matrix size of the images is 512 × 512, with slice thicknesses ranging from 0.45 to 6.00 mm, in-plane resolutions between 0.56 and 1.00 mm, and the number of slices per case ranging from 42 to 1026, totaling 58,638 slices [16]. The 3Dircadb dataset comprises contrast-enhanced abdominal CT scans from ten male and ten female subjects in the venous phase, with 15 cases featuring actual liver tumors.

For data allocation, 111 cases were used for training and validation, which were further divided into training and validation sets in a 3:1 ratio for model tuning. The remaining 20 cases were reserved as the test set to evaluate model performance. During evaluation, the model’s automatic segmentation results were compared with the ground truth annotations provided by radiation oncologists, enabling a quantitative assessment of segmentation accuracy and performance metrics.

The model training was performed using the Adam optimizer with a step decay learning rate schedule. The initial learning rate was set to 0.0001 [33], and it was reduced by 20% every 30 epochs. This strategy effectively mitigated instability caused by an excessively large learning rate while gradually decreasing the learning rate in the later training stages to facilitate fine-tuning of the network.

The experiments were conducted in a Windows 10 64-bit operating system environment, with hardware configurations including an Intel Xeon Gold 6132 processor (Intel, Santa Clara, CA, USA), 128 GB of RAM, and an NVIDIA TITAN RTX GPU (NVIDIA, Santa Clara, CA, USA). All experiments were implemented using Python 3.9 and the PyTorch 2.0 (GPU version) deep learning framework.

### 3.2. Comparison of the Different Loss Function

To clearly demonstrate the impact of the loss function on the segmentation results, the segmentation outcomes with the ELANRes-MSCA-UNet network using various loss functions were compared, and their performances were evaluated using the Dice value.

Based on the results presented in the Table 2, the findings indicate that the choice of loss function has a significant impact on the segmentation performance. For liver segmentation, HybridLoss1 (0.972) and HybridLoss2 (0.968) yielded comparatively good results, significantly outperforming DiceLoss (0.951), TverskyLoss (0.949), and BCELoss (0.958). These results suggest that hybrid loss functions effectively enhance liver segmentation’s accuracy by improving multi-scale feature modeling and boundary optimization capabilities.

In tumor segmentation, HybridLoss1 also demonstrated the best performance (0.729). The hybrid loss functions not only improved the liver segmentation but also performed well in segmenting small targets and complex tumor boundaries.

In conclusion, the hybrid loss function (HybridLoss1) exhibited high robustness in both liver and tumor segmentation tasks, substantially improving segmentation accuracy and emphasizing the importance of the loss function’s design in deep learning-based segmentation tasks. For future experiments, we will adopt HybridLoss1 as the loss function.

### 3.3. Ablation

To comprehensively evaluate the effectiveness of the ELANRes-MSCA-UNet network, we conducted ablation experiments to analyze the specific contributions of the MSCA module, the ELANRes module, and their combined use on model performance. The experiments included the following four model configurations: (a) the baseline model, UNet; (b) MSCA-UNet, incorporating only the MSCA module; (c) ELANRes-UNet, incorporating only the ELANRes module; and (d) the complete model, ELANRes-MSCA-UNet, which combines both the MSCA and ELANRes modules.

As shown in Table 3 and Table 4, the complete model, ELANRes-MSCA-UNet, achieved the best performance across all evaluation metrics for the liver segmentation task, with a *Dice* score of 0.972 and an *ASD* of 1.263, significantly outperforming the other models. Table 5 presents the results of ablation experiments on the LITS17 dataset, detailing the number of parameters, training time, and testing time. The results indicate that, while these metrics remain relatively unchanged with the addition of the modules, the segmentation performance notably improved. The improvement in performance with the ELANRes-MSCA-UNet model can be attributed to the complementary strengths of the MSCA and ELANRes modules. Specifically, the MSCA module enhances the model’s global feature extraction by capturing broad contextual information, which is critical for accurate liver segmentation. In contrast, the ELANRes module improves the segmentation boundaries, addressing the challenges in distinguishing between regions with similar intensities. MSCA-UNet improved the *Dice* score from 0.937 in the baseline model to 0.946 by enhancing global feature modeling capabilities, while ELANRes-UNet excelled in boundary optimization, reducing the *ASD* from 3.242 in the baseline model to 2.369. By combining both modules, the complete model not only leverages the strength of each individual module but also benefits from their interaction, leading to enhanced segmentation accuracy and finer boundary detail.

For the liver tumor segmentation task, the complete model, ELANRes-MSCA-UNet, demonstrated an exceptional performance, achieving a *Dice* score of 0.729 and an *ASD* of 1.012. It comprehensively outperformed other models in key metrics such as precision, recall, and *RVD*. These results can be attributed to the MSCA module’s ability to model global contextual information, which is particularly useful for detecting small tumors. The ELANRes module’s role in boundary refinement also plays a crucial part in improving the segmentation of tumor boundaries, especially when the tumor is surrounded by tissues with similar intensities. These results indicate that the complete model not only accurately captures target regions but also excels in optimizing boundary details and reducing false positive rates. The combination of these modules results in a balanced approach, improving both the detection of small target regions and the optimization of boundary details.

**Figure 5:**
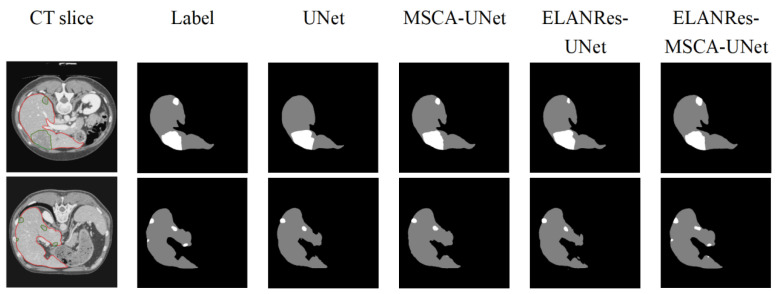
Qualitative segmentation results from the ablation experiment.

As shown in Figure 5, differences in the performances among the models in the segmentation results are clearly evident. The baseline model, UNet, performed reasonably well in the overall liver segmentation but exhibited noticeable missed and false segmentations in boundary details and tumor regions, particularly struggling to capture small tumors. The MSCA-UNet enhanced the global feature extraction and improved the tumor contour segmentation, but its ability to handle complex boundaries and small tumors remains limited. The ELANRes-UNet model improved the boundary optimization significantly, as it is designed to refine segment boundaries in regions with complex structures. However, it still struggles with the accurate segmentation of small tumors, as it is less effective in capturing fine-grained details across the entire image. In contrast, the complete model, ELANRes-MSCA-UNet, achieves a liver segmentation accuracy that is close to the ground truth (Label) and excels in boundary detection and detail refinement in tumor regions. It excels in small-tumor segmentation by leveraging both the global feature extraction of the MSCA module and the boundary refinement of the ELANRes module, showing remarkable improvements in both boundary accuracy and detection of small tumors, with a substantial reduction in missed segmentations.

In summary, the results of the ablation experiments demonstrate that the complete model, ELANRes-MSCA-UNet, effectively balances global modeling capability and local optimization by integrating the strengths of the MSCA and ELANRes modules. This integration significantly improves segmentation accuracy, enhances boundary optimization, and strengthens overall robustness, offering an efficient and reliable solution for medical image segmentation tasks in complex scenarios.

### 3.4. Comparison of Tumor Segmentation Performances Among Groups

In the tumor segmentation task, to further analyze the model’s performance in segmenting tumors of various sizes, the segmentation results were divided into two groups based on *Dice* scores: a high-performance group (*Dice* ≥ 0.6) and a low-performance group (*Dice* < 0.6). To enhance the reliability of the findings, additional control groups were introduced for comparison. For each group, the average major-axis length of the segmented liver tumors was calculated and compared across different models.

As shown in Table 6, the complete model, ELANRes-MSCA-UNet, achieved an average tumor major-axis length of 18.16 mm in the low-performance group, significantly outperforming the baseline model, UNet, which had an average length of 23.37 mm. This result highlights the distinct advantage of ELANRes-MSCA-UNet in segmenting small-sized tumor targets, for which precise boundary detection is essential. In comparison, MSCA-UNet, which incorporates only the MSCA module, performed better than UNet but still lagged behind ELANRes-MSCA-UNet, with an average length of 20.11 mm in the low-performance group. Similarly, ELANRes-UNet, incorporating only the ELANRes module, showed some improvement over UNet but still had an average axis length of 21.42 mm, indicating its limited capacity in segmenting smaller tumors without the benefit of the MSCA module.

In the high-performance group, the average tumor major-axis lengths for the two models were 41.83 mm and 44.72 mm, respectively, with relatively small differences. These findings demonstrate that while the complete model performs consistently well in handling large-sized tumors, it exhibits superior performance in segmenting small targets.

By incorporating multiple control groups, this comparison offers a more comprehensive assessment of model performance. The analysis reveals that while the MSCA-UNet enhances global feature extraction, which is particularly beneficial for segmenting larger tumors, it is less effective at capturing fine boundary details compared to the ELANRes-UNet. In contrast, the ELANRes-UNet excels at refining boundaries but faces challenges in accurately segmenting smaller tumors. The integration of both modules in the ELANRes-MSCA-UNet leads to a synergistic improvement, significantly enhancing both global feature extraction and boundary optimization. This combination results in more precise tumor segmentation, especially for small tumors.

In conclusion, ELANRes-MSCA-UNet, with its multi-scale modeling capability and boundary optimization mechanism, is particularly well-suited for complex scenarios involving tumors of varying sizes. Notably, in the segmentation of small-tumor targets, the model achieves higher accuracy and robustness.

### 3.5. Comparison with State-of-the-Art Methods

To evaluate the effectiveness and robustness of the ELANRes-MSCA-UNet network in liver and liver tumor segmentation tasks, we compared its performance with state-of-the-art semantic segmentation methods. We performed a subset of experiments using the same dataset and training strategies. All methods were implemented according to the model descriptions provided in the authors’ original papers.

Table 7 and Table 8 present the quantitative results of different models for liver and liver tumor segmentation. The data demonstrate that ELANRes-MSCA-UNet outperforms other models in liver segmentation, achieving the highest *Dice* score of 0.972, which represents a significant improvement. This highlights its exceptional performance in liver segmentation tasks. For the liver tumor segmentation, ELANRes-MSCA-UNet shows superior performance across several key metrics, including *Dice* (0.729) and precision (0.564), significantly surpassing other existing methods. These results indicate that ELANRes-MSCA-UNet is particularly effective in accurately capturing target regions in complex scenarios, excelling in reducing false positives and optimizing boundary details. Table 9 compares the five networks based on the parameter count, training time, and testing time. The results show that ELANRes-MSCA-UNet outperforms AttentionU-Net and ResUNet++ across three metrics. While R2U-Net and RIU-Net have minor advantages in some areas, our method delivers the best overall performance, affirming its superior capability in segmentation tasks.

**Figure 6:**
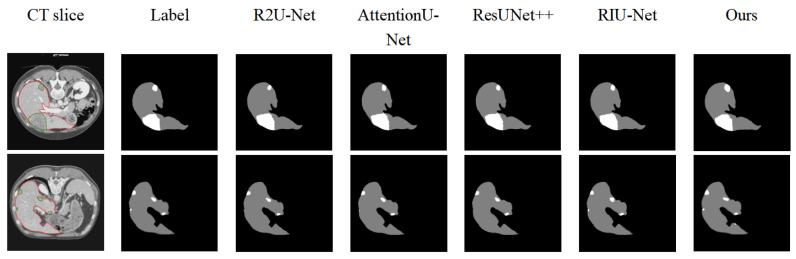
Comparison of the segmentation results for the different networks.

Figure 6 compares the visual segmentation results of five models. The proposed ELANRes-MSCA-UNet demonstrated a superior performance, particularly in segmenting both the liver and tumor regions, especially when the liver boundary contains tumors or when small tumors are challenging to segment. For instance, while R2U-Net and ResUNet++ can successfully segment large tumor regions near the liver boundary, they fail to detect smaller tumors adjacent to the liver. AttentionU-Net and RIU-Net can segment tumors of varying sizes, but they encounter issues with over-segmentation or incomplete segmentation. In contrast, ELANRes-MSCA-UNet provides the most accurate segmentation for both the liver and small tumors.

Overall, ELANRes-MSCA-UNet integrates the strengths of multi-scale contextual modeling and structural re-parameterization, surpassing other networks in segmentation accuracy while exhibiting greater robustness. These findings validate its effectiveness and demonstrate its advancement in medical image segmentation tasks.

## 4. Discussion and Conclusions

This paper proposes a 3D network architecture, ELANRes-MSCA-UNet, for automatic liver and liver tumor segmentation. By integrating the structurally re-parameterized residual module (ELANRes) and the multi-scale convolutional attention module (MSCA), the model demonstrates significant advantages in global feature modeling, boundary detail optimization, and small-target segmentation. The experimental results reveal that the proposed model outperformed the existing methods across multiple metrics, particularly excelling in small-target segmentation and its adaptability to complex scenarios. The specific advantages are as follows:

(1) Significant improvement in segmentation performance: ELANRes-MSCA-UNet achieved remarkable improvements in liver and liver tumor segmentation tasks, exhibiting outstanding performances in key metrics such as the *Dice* and *ASD*. The model accurately captures target boundaries, reduces false positives and missed segmentations, and provides an efficient solution for complex medical image segmentation tasks. In particular, the model’s innovation in boundary optimization allows it to excel in challenging segmentation scenarios, especially for small tumors or unclear boundaries.

(2) Robustness in small-target segmentation: Through the integration of multi-scale convolution and attention mechanisms, the model exhibits excellent robustness in small-tumor segmentation. Experiments demonstrate that ELANRes-MSCA-UNet significantly reduces missed segmentations of small targets and maintains consistent performance across tumor segmentation tasks of varying sizes, making it particularly effective in complex multi-scale segmentation scenarios. This robust performance is particularly valuable in clinical settings, where small or early-stage tumors are often difficult to detect.

(3) Innovative module design: The ELANRes module enhances feature extraction capabilities and reduces inference costs through structural re-parameterization technology, while the MSCA module captures global contextual information using multi-scale convolution and attention mechanisms. This design improves the model’s adaptability to low-contrast and blurred boundaries. Additionally, the weighted combination of multiple loss functions further enhances the segmentation accuracy, particularly in addressing class imbalance and recovering fine details. These innovations significantly enhance the model’s performance in medical imaging tasks, where the accurate segmentation of tumors with unclear boundaries is critical.

(4) Broad potential for clinical applications: ELANRes-MSCA-UNet offers reliable support for clinical tasks such as tumor volume assessment, growth trend analysis, and treatment planning. Its high-precision and robust segmentation results highlight the model’s broad potential for practical applications in medical image segmentation, making it highly suitable for complex clinical scenarios. In future clinical applications, the model could play significant roles in early diagnosis, personalized treatment planning, and post-treatment monitoring. Its ability to accurately segment even small or hard-to-detect tumors could enhance decision-making in real-time clinical settings, particularly in multi-modal imaging systems, such as the integration of CT, MRI, and PET data.

**Figure 7:**
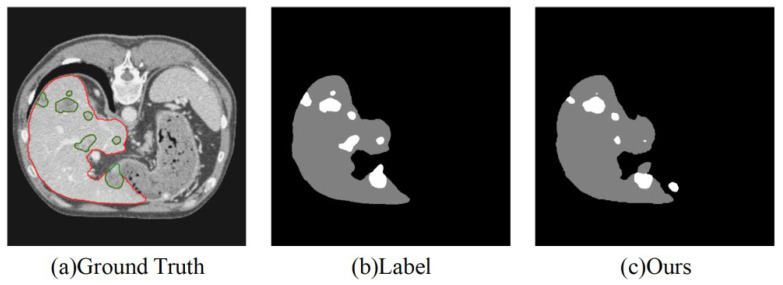
Performances in the liver and liver tumor segmentation tasks for small targets and complex scenarios.

Despite the excellent segmentation performance of our model, it still has the following limitations, as shown in Figure 7:

(1) Segmentation of very small tumors: The model may still miss segmenting very small tumors, primarily due to the weak feature representation of small targets. This limitation underscores the need for further refinement in the model’s ability to handle extremely small or low-contrast tumors. Future research should focus on improving the resolution of feature extraction in the network’s early layers, which could enhance the detection of minute lesions, particularly in regions with low contrast.

(2) Accuracy in multi-target segmentation: When the number of tumors is large and their distribution is complex, the segmentation performance may become unstable. Improving the model’s handling of multiple, closely spaced tumors is a key area for future improvement.

(3) High computational resource requirements: The network demands significant computational resources during the inference stage, which may limit its applicability in resource-constrained environments.

Future work will focus on optimizing the lightweight design of the network to improve the computational efficiency while enhancing its robustness in small-target and multi-target segmentation. These improvements aim to better adapt the model to more complex clinical application scenarios, including the integration of multi-modal imaging data and broader clinical scenarios. Efforts will also be directed toward improving the model’s generalizability to different imaging techniques and clinical environments.

## Figures and Tables

**Table 1:** Loss functions.

Loss Functions	Formula
DiceLoss	$Dice=\frac{2\cdot Intersection}{Union+\epsilon }$ DiceLoss=1−Dice
TverskyLoss	$TLoss=1-\frac{Intersection}{Intersection+\alpha \cdot FP+\beta \cdot FN+\epsilon }$
BCELoss	$BCELoss(y,\hat{y})=-\frac{1}{N}\sum_{i=1}^{N}\left[y_{i}\log({\hat{y}}_{i})+(1-y_{i})\log(1-{\hat{y}}_{i})\right]$
HybridLoss1	HLoss1=(1−λ)⋅DiceLoss+λ⋅BCELoss
HybridLoss2	HLoss2=(1−λ)⋅TLoss+λ⋅BCELoss

**Table 2:** Comparison of Dice values using various loss functions.

Loss Function	Data	Metrics	
		Dice (Liver)	Dice (Liver Tumor)
DiceLoss	LiTS	0.951	0.706
TverskyLoss	LiTS	0.949	0.705
BCELoss	LiTS	0.958	0.711
HybridLoss1	LiTS	0.972	0.729
HybridLoss2	LiTS	0.968	0.723

**Table 3:** Comparison of the ablation results for the liver segmentation.

Network	Data	Metrics	
		Dice	ASD
UNet	LiTS	0.937	3.242
MSCA-UNet	LiTS	0.953	2.891
ELANRes-UNet	LiTS	0.949	2.369
ELANRes-MSCA-UNet	LiTS	0.972	1.263

**Table 4:** Comparison of the ablation results for the liver tumor segmentation.

Network	Data	Metrics				
		Dice	ASD	RVD	Precision	Recall
UNet	LiTS	0.583	1.130	0.031	0.552	0.383
MSCA-UNet	LiTS	0.631	1.197	0.170	0.426	0.431
ELANRes-UNet	LiTS	0.620	1.260	0.192	0.387	0.427
ELANRes-MSCA-UNet	LiTS	0.729	1.012	0.021	0.564	0.508

**Table 5:** Parameters and time–cost of the ablation experiments.

Network	Data	Parameters	Time–Cost	
			Training Time	Test Time
UNet	LiTS	13,063,192	35 h 47 min	238 s
MSCA-UNet	LiTS	11,449,720	34 h 38 min	216 s
ELANRes-UNet	LiTS	14,738,072	36 h 08 min	251 s
ELANRes-MSCA-UNet	LiTS	13,124,600	35 h 51 min	240 s

**Table 6:** Evaluation of Tumor segmentation performance for different tumor sizes.

Network	Data	Average Major-Axis Length of Liver Tumors/mm	
		High-Performance Group	Low-Performance Group
UNet	LiTS	44.72	23.37
MSCA-UNet	LiTS	42.65	20.11
ELANRes-UNet	LiTS	42.95	21.42
ELANRes-MSCA-UNet	LiTS	41.83	18.16

**Table 7:** Comparison of the results for the liver segmentation with other state-of-the-art models.

Method	Data	Metrics	
		Dice	ASD
L. Bi et al. [21]	LiTS	0.934	258.598
Y. Yuan et al. [34]	LiTS	0.963	1.104
F. Isensee et al. [28]	LiTS	0.962	2.565
Z. Xu et al. [16]	LiTS	0.959	1.342
S. Chen et al. [16]	LiTS	0.954	1.386
R2U-Net [35]	LiTS	0.965	1.204
AttentionU-Net [36]	LiTS	0.962	1.375
ResUNet++ [37]	LiTS	0.954	1.437
RIU-Net [38]	LiTS	0.969	**1.102**
RHEU-Net [26]	LiTS	0.957	\
Ours	LiTS	**0.972**	1.263
Ours	3Dircadb	0.963	1.852

Note: Bold font indicates the best value for each metric.

**Table 8:** Comparison of the results for the liver tumor segmentation with other state-of-the-art models.

Method	Data	Metrics				
		Dice	ASD	RVD	Precision	Recall
L. Bi et al. [21]	LiTS	0.645	1.006	0.016	0.316	0.431
J. Zou et al. [16]	LiTS	0.702	1.189	5.921	0.148	0.479
X. Li et al. [17]	LiTS	0.686	1.073	5.164	0.436	**0.515**
X. Han et al. [19]	LiTS	0.674	1.118	−0.103	0.354	0.458
G. Chlebus et al. [39]	LiTS	0.676	1.143	0.464	0.519	0.463
D. Xu et al. [40]	LiTS	0.721	**0.896**	**−0.002**	0.549	0.503
R2U-Net [35]	LiTS	0.683	1.237	0.363	0.446	0.431
AttentionU-Net [36]	LiTS	0.702	1.068	0.217	0.524	0.504
ResUNet++ [37]	LiTS	0.679	1.176	0.874	0.501	0.465
RIU-Net [38]	LiTS	0.716	1.024	0.121	0.537	0.489
RHEU-Net [26]	LiTS	0.702	\	\	\	\
Ours	LiTS	**0.729**	1.012	0.021	**0.564**	0.508
Ours	3Dircadb	0.712	1.116	0.164	0.536	0.494

Note: Bold font indicates the best value for each metric.

**Table 9:** Comparison of the results for the parameters and time–cost with other state-of-the-art methods.

Network	Data	Parameters	Time-Cost	
			Training Time	Test Time
R2U-Net [37]	LiTS	9,778,818	34 h 57 min	227 s
AttentionU-Net [38]	LiTS	34,878,638	38 h 18 min	279 s
ResUNet++ [39]	LiTS	16,228,001	36 h 23 min	251 s
RIU-Net [40]	LiTS	2,389,474	34 h 21 min	210 s
Ours	LiTS	13,124,600	35 h 51 min	240 s

## Data Availability

The public datasets analyzed during the current study are available in the LiTS and 3Dircadb repositories: https://competitions.codalab.org/ and https://www.ircad.fr/research/data-sets/liver-segmentation-3d-ircadb-01/ (accessed on 1 March 2025).

## References

[1] Chhikara B.S., Parang K. (2023). Global Cancer Statistics 2022: The trends projection analysis. Chem. Biol. Lett..

[2] Luan S., Xue X., Ding Y., Luan S., Xue X., Ding Y., Wei W., Zhu B. (2021). Adaptive attention convolutional neural network for liver tumor segmentation. Front. Oncol..

[3] Tomoshige S., Oost E., Shimizu A., Watanabe H., Nawano S. (2014). A conditional statistical shape model with integrated error estimation of the conditions; application to liver segmentation in non-contrast CT images. Med. Image Anal..

[4] Wang X., Yang J., Ai D., Zheng Y., Tang S., Wang Y. (2015). Adaptive mesh expansion model (AMEM) for liver segmentation from CT image. PLoS ONE.

[5] Park H., Bland P.H., Meyer C.R. (2003). Construction of an abdominal probabilistic atlas and its application in segmentation. IEEE Trans. Med. Imaging.

[6] Xu Z., Burke R.P., Lee C.P., Baucom R.B., Poulose B.K., Abramson R.G., Landman B.A. (2015). Efficient multi-atlas abdominal segmentation on clinically acquired CT with SIMPLE context learning. Med. Image Anal..

[7] Wu W., Zhou Z., Wu S., Zhang Y. (2016). Automatic liver segmentation on volumetric CT images using supervoxel-based graph cuts. Comput. Math. Methods Med..

[8] Soler L., Delingette H., Malandain G., Montagnat J., Ayache N., Koehl C., Dourthe O., Malassagne B., Smith M., Mutter D. (2001). Fully automatic anatomical, pathological, and functional segmentation from CT scans for hepatic surgery. Comput. Aided Surg..

[9] Linguraru M.G., Richbourg W.J., Liu J., Watt J.M., Pamulapati V., Wang S., Summers R.M. (2012). Tumor burden analysis on computed tomography by automated liver and tumor segmentation. IEEE Trans. Med. Imaging.

[10] Moltz J.H., Bornemann L., Kuhnigk J.M., Dicken V., Peitgen E., Meier S., Bolte H., Fabel M., Bauknecht H.C., Hittinger M. (2009). Advanced segmentation techniques for lung nodules, liver metastases, and enlarged lymph nodes in CT scans. IEEE J. Sel. Top. Signal Process..

[11] Massoptier L., Casciaro S. (2008). A new fully automatic and robust algorithm for fast segmentation of liver tissue and tumors from CT scans. Eur. Radiol..

[12] Conze P.H., Noblet V., Rousseau F. (2017). Scale-adaptive supervoxel-based random forests for liver tumor segmentation in dynamic contrast-enhanced CT scans. Int. J. Comput. Assist. Radiol. Surg..

[13] Minaee S., Boykov Y., Porikli F. (2021). Image segmentation using deep learning: A survey. IEEE Trans. Pattern Anal. Mach. Intell..

[14] Chen C., Zhou K., Wang Z., Zhang Q., Xiao R. (2023). All answers are in the images: A review of deep learning for cerebrovascular segmentation. Comput. Med. Imaging Graph..

[15] Christ P.F., Elshaer ME A., Ettlinger F., Tatavarty S., Bickel M., Bilic P., Rempfler M., Armbruster M., Hofmann F., D’Anastasi M. (2016). Automatic liver and lesion segmentation in CT using cascaded fully convolutional neural networks and 3D conditional random fields. Medical Image Computing and Computer-Assisted Intervention–MICCAI 2016, Proceedings of the 19th International Conference, Athens, Greece, 17–21 October 2016.

[16] Bilic P., Christ P., Li H.B., Vorontsov E., Ben-Cohen A., Kaissis G., Szeskin A., Jacobs C., Mamani G.E.H., Chartrand G. (2023). The liver tumor segmentation benchmark (lits). Med. Image Anal..

[17] Li X., Chen H., Qi X., Dou Q., Fu C.W., Heng P.A. (2018). H-DenseUNet: Hybrid densely connected UNet for liver and tumor segmentation from CT volumes. IEEE Trans. Med. Imaging.

[18] Dey R., Hong Y. Hybrid cascaded neural network for liver lesion segmentation. Proceedings of the 2020 IEEE 17th International Symposium on Biomedical Imaging (ISBI).

[19] Han X. (2017). Automatic liver lesion segmentation using a deep convolutional neural network method. arXiv.

[20] Gruber N., Antholzer S., Jaschke W., Kremser C., Haltmeier M. A joint deep learning approach for automated liver and tumor segmentation. Proceedings of the 2019 13th International conference on Sampling Theory and Applications (SampTA).

[21] Bi L., Kim J., Kumar A., Feng D. (2017). Automatic liver lesion detection using cascaded deep residual networks. arXiv.

[22] Kaluva K.C., Khened M., Kori A., Krishnamurthi G. (2018). 2D-densely connected convolution neural networks for automatic liver and tumor segmentation. arXiv.

[23] Deng Z., Guo Q., Zhu Z. (2019). Dynamic regulation of level set parameters using 3D convolutional neural network for liver tumor segmentation. J. Healthc. Eng..

[24] Liu T., Liu J., Ma Y., He J., Han J., Ding X., Chen C.T. (2021). Spatial feature fusion convolutional network for liver and liver tumor segmentation from CT images. Med. Phys..

[25] Zhu F., Niu Q., Li X., Zhao Q., Su H., Shuai J. (2024). FM-FCN: A neural network with filtering modules for accurate vital signs extraction. Research.

[26] Sun L., Jiang L., Wang M., Wang Z., Xin Y. (2024). A Multi-Scale Liver Tumor Segmentation Method Based on Residual and Hybrid Attention Enhanced Network with Contextual Integration. Sensors.

[27] Ma J., He Y., Li F., Han L., You C., Wang B. (2024). Segment anything in medical images. Nat. Commun..

[28] Isensee F., Jaeger P.F., Kohl S.A.A., Petersen J., Maier-Hein K.H. (2021). nnU-Net: A self-configuring method for deep learning-based biomedical image segmentation. Nat. Methods.

[29] Ding X., Zhang X., Ma N., Han J., Ding G., Sun J. Repvgg: Making vgg-style convnets great again. Proceedings of the IEEE/CVF Conference on Computer Vision and Pattern Recognition.

[30] Shao H., Zeng Q., Hou Q., Yang J. (2023). MCANet: Medical Image Segmentation with Multi-Scale Cross-Axis Attention. arXiv.

[31] Guo M.H., Lu C.Z., Hou Q., Liu Z., Cheng M.M., Hu S.M. (2022). Segnext: Rethinking convolutional attention design for semantic segmentation. Adv. Neural Inf. Process. Syst..

[32] Vorontsov E., Tang A., Pal C., Kadoury S. Liver lesion segmentation informed by joint liver segmentation. Proceedings of the 2018 IEEE 15th International Symposium on Biomedical Imaging (ISBI 2018).

[33] Goodfellow I. (2016). Deep Learning.

[34] Yuan Y. (2017). Hierarchical convolutional-deconvolutional neural networks for automatic liver and tumor segmentation. arXiv.

[35] Alom M.Z., Hasan M., Yakopcic C., Taha T.M., Asari V.K. (2018). Recurrent residual convolutional neural network based on u-net (r2u-net) for medical image segmentation. arXiv.

[36] Oktay O., Schlemper J., Folgoc L.L., Lee M., Heinrich M., Misawa K., Mori K., McDonagh S., Hammerla N.Y., Kainz B. (2018). Attention u-net: Learning where to look for the pancreas. arXiv.

[37] Jha D., Smedsrud P.H., Johansen D., De Lange T., Johansen H.D., Halvorsen P., Riegler M.A. (2021). A comprehensive study on colorectal polyp segmentation with ResUNet++, conditional random field and test-time augmentation. IEEE J. Biomed. Health Inform..

[38] Lv P., Wang J., Wang H. (2022). 2.5 D lightweight RIU-Net for automatic liver and tumor segmentation from CT. Biomed. Signal Process. Control.

[39] Chlebus G., Meine H., Moltz J.H., Schenk A. (2017). Neural network-based automatic liver tumor segmentation with random forest-based candidate filtering. arXiv.

[40] Yang D., Xu D., Zhou S.K., Georgescu B., Chen M., Grbic S., Metaxas D., Comaniciu D. (2017). Automatic liver segmentation using an adversarial image-to-image network. Medical Image Computing and Computer Assisted Intervention—MICCAI 2017, Proceedings of the 20th International Conference, Quebec City, QC, Canada, 11-13 September 2017.

